# Supplementary material for: A novel benchmark for COVID-19 pandemic testing effectiveness enables the accurate prediction of new Intensive Care Unit admissions
Source: Sci Rep. 2021 Oct 13;11:20308. doi: 10.1038/s41598-021-99543-y (PMC8514432; doi:10.1038/s41598-021-99543-y)
Supplement: Supplementary file 1 — Supplementary Information 1. [file 41598_2021_99543_MOESM1_ESM.docx]

**Supplementary Charts and Tables for the assessment of the predictive potential of the ICU Rate metric.**

**A**

**Figure 1A-S.** *Comparison of trendlines of ICU rate, Severity Detection Rate, Daily needs for new ICU admissions, Positivity rate, and number of Daily Tests, in the period from 7/5/2020 to 8/8/2021. The Daily needs for new ICU admissions and the number of Daily Tests represent rolling 7-day averages. ICU Rates, Severity Detection Rates and Positivity Rates were calculated from the rolling 7-day averages of their components. All numbers were normalized by their maximum value in the examined period.*

**B**

**Figure 1Β-S.** *Comparison of trendlines of ICU rate, Severity Detection Rate and Daily ICU needs in the period from 20/8/2020 to 8/8/2021. The Daily ICU needs represent rolling 7-day averages. ICU Rates and Severity Detection Rates were calculated from the rolling 7-day averages of their components. All numbers were normalized by dividing with their average value in the examined period.*

**Figure 2-S.** *Correlation between the ICU Rate and rolling 7-day averages of the number of daily tests, with Spearman r = -0.37, p = 10 ^-16^, N = 451. Numbers of daily tests derived from the period from 15/5/2020 to 8/8/2021.*

**Figure 3-S.** *Correlation of the numerator and denominator of IR, i.e., number U versus the number of cases_t-12_ with a lag of 12 days. Numbers of detected cases were derived from the period from 17/10/2020 to 8/8/2021.*

**A**

**B**

**Figure 4-S.** (A) *Correlation between observed and predicted daily needs for new ICU admissions for the period between 17/10/2020 and 8/8/2021 employing Severity Detection Rate regression equations, with Pearson* r = 0.98, p = 10^-223^, RMSE = 5.82; with n = 296, observed U[max] = 125, U[average] = 51. *(B) The respective time-series plots for visual inspection of the fit.*

**Table 1-S.** *Characterization of the ICU Rate, Severity Detection Rate and the number of daily tests for each of the 6 time-intervals of distinct testing levels in Greece (2^nd^ pandemic wave is included in the third interval).*

**Table 2-S.** Regression equations per distinct period of stable ICU Rate and Severity Detection Rate, with respective average numbers of observed daily needs for new ICU admissions, cases detected and tests performed, vaccination coverage at the beginning of each period, percent of Delta variant prevalence in cases detected, along with important dates and comments that potentially influenced the course of the pandemic in Greece (each period is color-coded with reference to the respective ‘distinct periods of testing levels’, in Table 1-S).
